# Comparison of two commercial methods for smooth-shelled mussels (*Mytilus* spp.) species identification

**DOI:** 10.1016/j.fochms.2022.100121

**Published:** 2022-07-14

**Authors:** Cynthia M. Asorey, Felipe Jilberto, Ilka Haase, Rainer Schubbert, María Angélica Larraín, Cristián Araneda

**Affiliations:** a Food Quality Research Center. Universidad de Chile, Santiago, Chile; b Universidad Católica del Norte, Facultad de Ciencias del Mar, Sala de Colecciones Biológicas, Departamento de Biología Marina, Larrondo 1281, Coquimbo, Chile; c Universidad de Chile, Facultad de Ciencias Agronómicas, Departamento de Producción Animal, Avenida Santa Rosa 11315, La Pintana, Santiago, Chile; d Universidad de Chile, Facultad de Ciencias Químicas y Farmacéuticas, Departamento de Ciencia de los Alimentos y Tecnología Química, Santiago, Chile; e Eurofins Genomics, Anzinger Str. 7a, 85560 Ebersberg, Germany

**Keywords:** Seafood traceability, H1C gene, HRM, PAPM, FINS, DNA sequence analysis

## Abstract

Seafood international trade has increased the labeling requirements in standards and regulations to include product information that enable traders and consumers to make informed choices. The European Union (EU) Regulation No. 1379/2013 imposes the declaration of an official commercial designation and scientific names for all the fishery and aquaculture products to be offered for sale to the final consumers. DNA analyses are used to enforce this regulation and to test authenticity in processed foods. We compared the performance of two mono-locus approaches for species identification (SI) in 61 *Mytilus* mussels: the high-resolution melting analysis of the *polyphenolic adhesive protein* gene and the partial sequencing of the *histone H1C* gene. The *H1C* sequences were analyzed with five different methods. Both approaches show discrepancies in the identification of putative hybrids (0.0 < κ < 0.687 and 0.0 < MCC < 0.724). Excluding putative hybrids, methods show substantial to perfect agreement (0.772 < κ < 1.0 and 0.783 < MCC < 1.0). This study highlights the need to use standardized molecular tools, as well as to use multi-locus methods for SI of *Mytilus* mussels in testing laboratories.

## Highlights

• A comparison of the sequence analysis of the H1C gene and HRM analysis of the PAP gene for species identification in the Mytilus genus was performed.
• Sequence analysis of the H1C gene affects the performance of species identification.
• Sequence analysis H1C reveals hybrids not detected by the HRM-PAPM.
• Both methods show perfect agreement when putative hybrids are excluded from the analysis.

## 1 Introduction

Smooth-shelled mussels (*Mytilus* spp.) includes, at least, seven species with statistics in FAO 2019 database. Among these edible mussels, 41.5 %, 35.7 % and 14.6 % of the production came from *Mytilus chilensis*, *M. edulis* and *M. galloprovincialis*, respectively (FAO, 2021). It is well known that where more than one *Mytilus* species coexists in the same geographic area, they hybridize posing challenges to species identification (SI) (Coustau et al., 1991, Michalek et al., 2016). Seafood international trade increased requirements regarding quality, safety, authenticity and sustainability production in food standards and regulations to give confidence to regulators, food traders and consumers, and to enable them to make informed choices (Codex Alimentarius, 2020, EU No /n.d.1224, 2009, EU. No/1380, 2013, 2013, EU.No/1379, 2013, 2013). These regulations require traceability “from ocean to fork”, that means among others, to identify the species to which the individuals belong (Ogden et al, 2008). For example, regulation EU No.1379/2013 establishes labelling requirements for marketed seafood and foodstuffs, such as declaring the commercial designation and scientific name of the species in the product labels (D’Amico et al., 2016, Tinacci et al., 2019). To enforce this regulation, methods based on DNA analysis have been employed to investigate intentional and unintentional species substitution in seafood (Fernández-Tajes et al., 2011, Santaclara et al., 2006, Verrez-Bagnis et al., 2018).

The most popular approach to specimen identification is sequencing analysis, which can be performed by different methods. One of them is the Forensically Informative Nucleotide Sequencing (FINS), which involves the estimation of sequence similarity among specimens by phylogenetic methods (i.e. Kimura-2, Tamura-Nei or Jukes-Cantor) based on genetic distances and drawing a phylogenetic tree using UPGMA or Neighbor-Joining (NJ) algorithms (Bartlett & Davidson, 1992). However, methodologies based on tree topologies perform poorly for specimen identification, therefore alternative approaches based on direct sequence comparison and genetic distances have been proposed (Collins & Cruickshank, 2013). One of these approaches is the automatic barcoding gap discovery (ABGD) for primary species delimitation (Puillandre, Lambert, Brouillet, & Achaz, 2012). ABGD begins detecting the first significant gap and uses it to partition the data. Next, limit and gap detection inferences are recursively applied to previously obtained groups to get finer partitions until no further partitioning is possible (Puillandre et al., 2012). Another approach is the “best close match“ (BCM), where a specimen is successfully identified if its sequence shows the smallest genetic distance to all conspecific sequences, and it is within the 95^th^ percentile of all intraspecific distances (Meier, Shiyang, Vaidya, & Ng, 2006). This strategy requires determining the threshold similarity value that defines how similar a barcode match needs to be before it can be identified (Meier et al., 2006). Finally, the “all barcodes” (AB) method is a more rigorous application of the best close match strategy (Meier et al., 2006). It uses information from all conspecific barcodes in the database, instead of just focusing on the most similar ones. The barcodes are sorted by similarity to the unknown sample using the same threshold as for the best close match. The identification is achieved if at least two sequences of the query species are available in the database and when all conspecific barcodes topped the list of the best matches. This method is more confident about assigning a species name to a query in cases where multiple species names are found on the list of best matches (Meier et al., 2006). Specifically, for the genetic identification of the three *Mytilus* species found in Europe (*M. edulis, M. trossulus* and *M. galloprovincialis*), Inoue, Waite, Matsuoka, Odo, and Harayama (1995) developed a PCR-length polymorphism analysis from the nonrepetitive region in the polyphenolic adhesive protein gene (*PAP*). This region is an attractive target as it contains SNP and size polymorphisms between species within the *Mytilus* genus. Later, to differentiate between *M. chilensis* and *M. galloprovincialis,* Santaclara et al. (2006) added a Restriction Fragment Length Polymorphism analysis (PCR-RFLP) of the same amplicon. Always targeting this gene, Jilberto, Araneda, and Larraín (2017) developed a High-Resolution Melting (HRM) analysis (HRM-PAPM) to identify *M. chilensis, M. galloprovincialis, M. edulis* and their F_1_ hybrids. By the HRM analysis is possible to obtain the genotypes of single nucleotide polymorphisms (SNPs) and detects length polymorphisms in small amplicons, showing distinguishable melting curves, permitting discrimination among species (Verrez-Bagnis et al., 2018). The HRM-PAPM analysis allows for distinguish, *M. galloprovincialis* (genotype GG, amplicon size 116 bp), *M. edulis* (genotype GG, 170 bp), and *M. chilensis* (genotype TT, 116 bp) (Jilberto et al., 2017). HRM is ideal for laboratory analysis because is fast, accurate and less expensive in comparison with other DNA based methodologies (Jilberto et al., 2017). Another method used in the identification of *Mytilus* species is the comparison of the partial sequence of the histone *H1C* gene (Eirín-López, González-Tizón, Martinez, & Méndez, 2002). Specifically, *H1C* gene is used to separate *M. chilensis* from other *Mytilus* species (*M. galloprovincialis, M. edulis,* and *M. trossulus)*. Both, the HRM-PAPM method and the *H1C* sequencing are easy to apply in routine testing to identify *Mytilus* species in traded seafood. However, basing SI on the analysis of a single gene, known as the mono-locus approach, can give contradictory results due to differences in the evolutionary rate of the analyzed genes (Larraín et al., 2019, Väinölä and Strelkov, 2011). Also, not all markers can differentiate among all species (Larraín et al., 2019). To overcome these problems related to the analysis of a single marker, the multi-locus analysis integrating genomic information from SNP panels to identify *M. trossulus*, M. *galloprovincialis*, *M. edulis*, and *M. chilensis* has been used by Larraín et al., 2018, Wenne et al., 2022 with 49 and 54 SNPs, respectively.

In this work, we tested and compared the performance of the HRM-PAPM method and the *H1C* sequence analysis for species identification in smooth-shelled mussels *Mytilus galloprovincialis* and *M. chilensis* using individuals previously identified with a panel of 49 SNPs.

## 2 Material and methods

### 2.1 Mussel samples, DNA extraction and species identification

Fifty-two samples of the *Mytilus* mussels had obtained from six growing centers in the Reloncaví Sound area (−41.700, −72.833), where the Chilean mussel aquaculture industry is located and one in Peel Island (−50.842, −74.011). Nine fresh individuals were obtained from a growing center in the Dichato Bay (−36.538, −72.957), 570 km away from the aquaculture area. Reloncaví Sound and Peel Island samples were collected in 2009 and 2013 (n = 26 per year), and Dichato samples were collected in 2009 (Table S1). All samples were processed fresh up to 24 h after collection, dissected and approximately 200 mg of mantle edge tissue was fixed with 95 % ethanol and stored at −20 °C until DNA extraction. DNA was obtained by the phenol–chloroform method, adapted for mussels (Larraín, Díaz, Lamas, Vargas, & Araneda, 2012). The DNA concentration was estimated with a NanoDrop ND-2000 spectrophotometer (Thermo Fischer Scientific). DNA integrity was assessed using agarose gel electrophoresis (0.7 % w/v). Mussel species were previously determined with a 49 SNPs panel by Larraín et al. (2018), resulting in nine as *M. galloprovincialis* (Mg) and 52 as *M. chilensis* (Mch).

### 2.2 High resolution melting (HRM-PAPM) analysis

The HRM-PAPM analysis was performed in an Eco Real-Time PCR System 4.0 (Illumina®) and Mic qPCR Cycler (Bio Molecular Systems). In all HRM analyses, *M. galloprovincialis*, *M. edulis*, and *M. chilensis* reference samples were included as controls*.* Also, negative control without DNA was included in all runs. Species identification was performed through clearly distinguishable melting curves, for extended protocol see Jilberto et al. (2017). The validation of the HRM-PAPM analysis was published in Quintrel et al. (2021), and a validation summary is included in Table S2.

### 2.3 Histone H1C gene sequencing

A partial sequence of approximately 400 bp of the *H1C* gene was amplified by PCR using the primer set H1CF (5′-CATCATGGCCAACTTCAACG-3′) and H1CR (5′-GGCTGAATAGCCTCTGCAGA-3′) (Pérez-García, Morán, & Pasantes, 2014). The final product length was checked by electrophoresis on a 2 % agarose gel. PCR and sequencing were performed in the facilities of Eurofins Genomics GmbH (Ebersberg, Germany). All reactions were carried out in a 25-µL volume containing 1.5 U AmpliTaq Gold DNA Polymerase (Applied Biosystems®), 2.5 µL 10 × Gold Star Buffer (Promega Corporation®), 1 to 5 ng DNA and 0,2 µM of each primer). The PCR reaction was performed on a GeneAmp 9700 thermocycler (Applied Biosystems®). Initial denaturation was performed at 95 °C for 12 min; followed by 15 cycles of denaturation at 96 °C for 25 s, annealing at 55 °C for 20 s, and extension at 72 °C for 30 s; followed by 25 cycles of denaturation at 96 °C for 20 s, annealing at 58 °C for 20 s, and extension at 72 °C for 30 s with a final elongation step at 72 °C for 3 min. Double-stranded PCR amplicons sequencing was performed in an ABI 3130xl genetic analyzer (Applied Biosystems®).

#### 2.3.1 Forensically informative nucleotide sequencing (FINS)

The 61 obtained sequences (GenBank accession numbers MT949777 to MT949837) were aligned together with 14 other *Mytilus* spp. *H1C* partial sequences recovered from GenBank (Drabent et al., 1999, Eirín-López et al., 2002) and two unpublished sequences used by Eurofins (Table 1), with the MAFFT 7.388 plugin (Katoh & Standley, 2013) in Geneious 11.5 (BiomattersL) and manually edited. Phylogenetic analysis was carried out with Geneious tree builder module. A neighbour-joining phylogenetic tree based on the Tamura-Nei model genetic distances (Verrez-Bagnis et al., 2018) was constructed with 10,000 bootstrap replicas as a reliability test (Terol, Mascarell, Fernandez-Pedrosa, & Pérez-Alonso, 2002) using *M. californianus* as outgroup (AJ416421) because this species was the most distant taxa within *Mytilus* genus as revealed by these sequences. Species were identified when the individual clusters with conspecific barcodes (Meier et al., 2006).

**Table 1:** *Mytilus* spp. *H1C* partial sequences used in the sequence analysis.

ID Genbank	Declared species	N° of sequences	Reference
AJ416421	*M. californianus*	1	Eirín-López et al. (2002).
AJ416422	*M. chilensis*	1	Eirín-López et al. (2002).
–	*M. chilensis*	1	EUROFINS Genomics, Ebersberg, Germany
MT949777 to MT949820	*M. chilensis*	44	Current work
MT949830 to MT949837	*M. chilensis*	8	Current work
AJ416423	*M. edulis*	1	Eirín-López et al. (2002).
AJ224069 to AJ224077	*M. edulis*	9	Drabent et al. (1999).
–	*M. edulis*	1	EUROFINS Genomics, Ebersberg, Germany
AJ416424	*M. galloprovincialis*	1	Eirín-López et al. (2002).
MT949821 to MT949828	*M. galloprovincialis*	9	Current work
AJ416425	*M. trossulus*	1	Eirín-López et al. (2002).
	**Total**	**77**	

#### 2.3.2 Direct sequence comparison (DSC)

The raw chromatograms of the 61 individuals were manually edited in Geneious 11.5 (Biomatters) and aligned as described in FINS analysis (2.3.1). According to this alignment analysis, the species of *M. chilensis* and *M. galloprovincialis* individuals were assigned considering the genotype at polymorphic sites (single or double pick in chromatograms) shared between both species. In this case, individuals heterozygous showing double picks in these sites were considered as putative hybrids. Therefore, two datasets were defined: the first one (dataset1) containing sequences from all the 61 individuals of the *Mytilus* genus sampled in this study and described in *2.1.* The second dataset (dataset2) contains the abovementioned sequences, excluding the 12 putatively hybrid individuals determined from the analysis of the raw chromatograms (see Table 2 and results in 3.2.2). An individual was assigned to a species when the percentage of similarity of its *H1C* sequence was greater than 98 % compared with the other conspecific sequences (Armani et al., 2015, Barbuto et al., 2010, Hebert et al., 2003). A summary of the validation for the DSC analysis on the *H1C* gene is included in Table S2.

**Table 2:** Polymorphisms sites in the *H1C* sequence from *Mytilus* species and putative hybrids *Mytilus chilensis* (Mch) × *M. galloprovincialis* (Mg). The double picks registered in several putative hybrids are in red (Y = C/T, R = G/A, M = C/A).

Individual	GenBank ID	115	151	193	259	322
Putative hybrid *Mch* × *Mg*	MT949835	Y	G	T	A	C
Putative hybrid *Mch* × *Mg*	MT949831	Y	R	Y	R	M
Putative hybrid *Mch* × *Mg*	MT949833	Y	G	T	A	C
Putative hybrid *Mch* × *Mg*	MT949837	Y	R	C	R	M
Putative hybrid *Mch* × *Mg*	MT949834	Y	G	T	A	C
Putative hybrid *Mch* × *Mg*	MT949795	Y	R	Y	R	M
Putative hybrid *Mch* × *Mg*	MT949829	Y	R	T	R	M
Putative hybrid *Mch* × *Mg*	MT949832	Y	G	Y	R	M
Putative hybrid *Mch* × *Mg*	MT949830	Y	R	Y	A	M
Putative hybrid *Mch* × *Mg*	MT949836	Y	R	Y	R	M
Putative hybrid *Mch* × *Mg*	MT949844	Y	R	Y	R	M
*M. chilensis*	MT949794	C	G	T	A	C
*M. chilensis*	MT949778	C	G	T	A	C
*M. chilensis*	MT949814	C	G	T	A	C
*M. galloprovincialis*	MT949827	T	A	C	G	A
*M. galloprovincialis*	MT949823	T	A	C	G	A
*M. galloprovincialis*	MT949824	T	A	C	G	A
*M. chilensis*	Eurofins	C	G	T	A	C
*M. edulis*	Eurofins	T	A	C	G	A
*M. trossulus*	AJ416425	A	A	C	A	C
*M. galloprovincialis*	AJ416424	T	A	C	G	A
*M. californianus*	AJ416421	A	A	C	A	T

#### 2.3.3 Other sequence analysis methods

Also, we tested the other three methods commonly used in sequence comparison analysis to identify *M. chilensis* and *M. galloprovincialis* individuals. First, the ABGD method was run 4 times from its webpage: https://bioinfo.mnhn.fr/abi/public/abgd/abgdweb.html with both evolution models (Jukes-Cantor JC69 and Kimura 2-parameter K80) and a Prior Intraspecific divergence value between 0.1 and 0.001 (Puillandre et al., 2012). The second and third methods were the BCM and the AB, both applied to estimate a threshold similarity value to consider individuals as conspecific (Meier et al., 2006)¡. They were run twice in the TaxonDNA/Species identifier ver. 1.8 software with Kimura 2-parameters correction using the two previously described datasets (Meier et al., 2006).

### 2.4 Agreement between the HRM-PAPM and histone H1C sequence analysis

The classification agreement between the HRM-PAPM and each of the *Histone H1C* sequence analyses described in 2.3 (DSC, BCM, AB and ABGD methods), was evaluated using individuals from dataset1 and dataset2. We calculate Cohen's Kappa coefficient (κ) and the Matthews correlation coefficient (MCC) to evaluate the quality of SI obtained by the different molecular assays. κ statistic measures the agreement between two methods that classify items into mutually exclusive categories (Rotondi, 2018), and it was estimated with the 95 % confidence interval (Cohen, 1960), using the “fmsb” R package version 0.6.3 (Nakasawa, 2018). FDR correction was applied to κ p-values to avoid the alpha-inflation produced by multiple testing (García, 2004). MCC is a measure of the quality of classification agreement between two categorical variables (Matthews, 1975) that was estimated using the “mltools” R package version 0.3.5.

## 3 Results

### 3.1 HRM-PAPM analysis

This method based on the melting curves from the 61 samples, classified nine individuals as *M. galloprovincialis* and the remaining 52 as *M. chilensis*, without any evidence of hybridization (Figure S1).

### 3.2 Histone H1C gene sequence analysis

#### 3.2.1 Fins

**Fig. 1:**
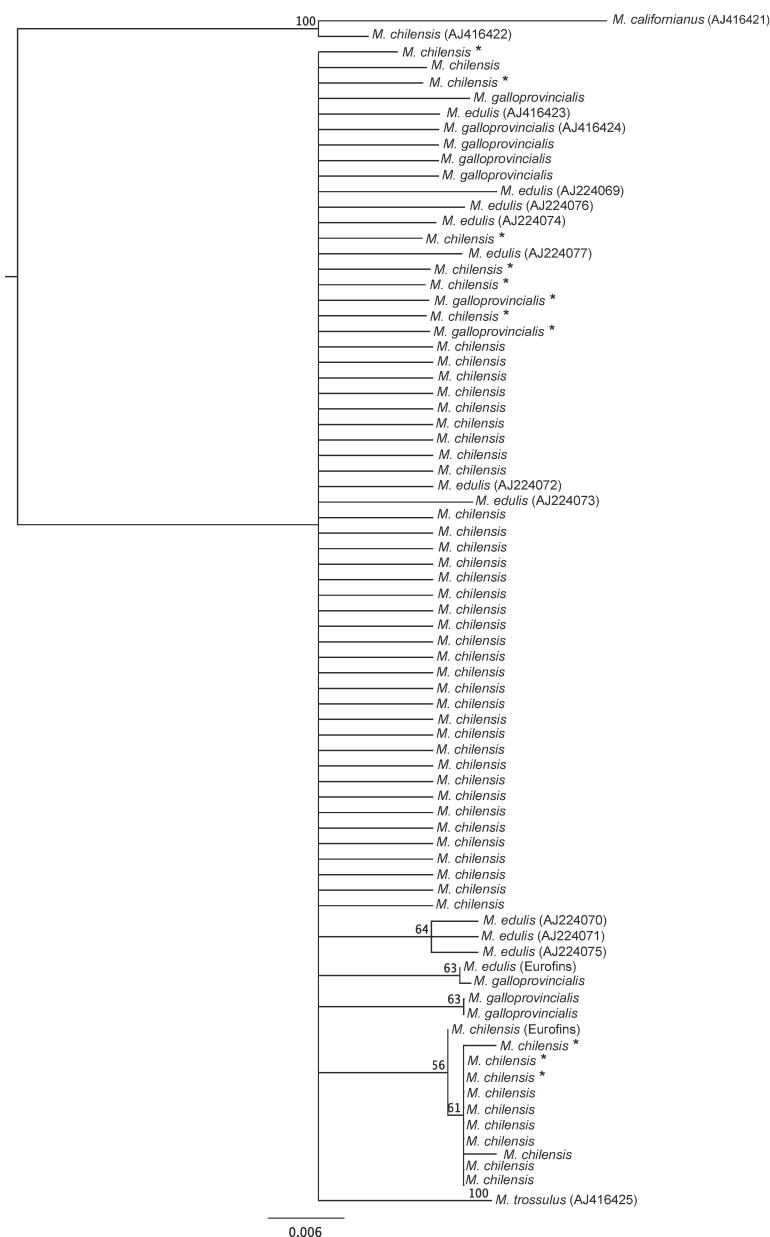
Neighbour-joining tree of the partial sequence of the *HC1* gene of the 71 *Mytilus* sequences analyzed. GenBank downloaded sequences are identified by their accession number and the two unpublished sequences used by Eurofins. The twelve putative hybrids are indicated by (*).

The phylogenetic tree shows two principal clades (Fig. 1), the first one containing only two individuals from data uploaded by Eirín-López et al. (2002) on GenBank as *M californianus* (AJ416421), and *M. chilensis* (AJ416422). The second clade grouped the remaining 75 individuals, among them, only *M. trossulus* was separated significantly with *H1C* gene analysis. The separations within this clade did not reach the minimum threshold confidence to be considered significant.

#### 3.2.2 DSC

After aligning the *H1C* 397 bp amplicon from all sequences available from GenBank and this work, 49 polymorphic sites were found. Despite these polymorphic sites, all sequences showed at least 91.44 % of similarity (Tables S3 and S4)*.* The lowest similarity value between *M. galloprovincialis* and either *M. edulis* (download from GenBank) or *M. chilensis* was 97.73 %*.* (Table S4). Besides, in some cases, sequences corresponding to *M. edulis* and *M. galloprovincialis* were identical (Table S3).

Twelve individuals (GenBank accession numbers MT949820, MT949828 to MT949837 and MT949795) showed chromatograms curves with double peaks at five sites. These sites were: 115, 151, 193, 259, 322 (Table 2). In *M. galloprovincialis* these sites corresponded to T, A, C, G and A respectively, whereas in *M. chilensis* were C, G, T, A and C, respectively. The 12 individuals that presented double peaks in these five sites, showed the two nucleotides corresponding to the polymorphisms found between *M. chilensis* and *M. galloprovincialis* mentioned above, and were considered heterozygous for these sites and putative hybrids.

The sequence matrix showed a 97.73 % to 98.74 % of similarity between *M. chilensis* and *M. galloprovincialis* (Table S3 and S4). Considering the twelve sequences of putative hybrids carrying double picks in the five sites, the similarity with *M. chilensis* sequences ranged from 98.36 % to 99.87 % and with *M. galloprovincialis* ranged from 97.86 % to 99.37 % (Tables S3 and S4). Therefore, pairwise similarities among individuals from both species and their putative hybrids were higher than 97.73 % (Table S3 and S4).

Using dataset2 (excluding individuals whose sequences showed double picks) DSC analysis successfully assigned all samples to the correct species when a sequence similarity threshold over 99 % was considered.

#### 3.2.3 Other sequence analysis methods

The ABGD method was not able to separate the individuals from dataset1 into groups according to the nominal species classification, the prior intraspecific distance ranged between 0.0028 and 0.0077. The BCM and the AB methods calculated a threshold similarity value of 1.28 %. BCM analysis correctly recognized the species from 55 sequences (90.16 %), four (6.56 %) were ambiguous (MT949829, MT949830, MT949832 and MT949837) and two (3.28 %) were incorrectly classified (MT949828 and MT949836). The ambiguous or incorrectly classified sequences corresponded to putative hybrids found in the DSC analysis. However, six sequences of those individuals who presented double picks (MT949795, MT949820, MT949831, MT949833, MT949834 and MT949835) were classified as *M. chilensis.* The AB method classified all the 61 individuals from dataset1 as ambiguous (Table 3).

**Table 3:** Performance of sequence analysis methods applied to species identification based on Histone *H1C* gene for the dataset1.

	Individuals identified [%]		
SI method	Correctly	Ambiguous	Incorrectly
FINS	0	100	0
DSC	80.3	19.7	0
ABGD	0	100	0
BCM	88.5	8,2	3.3
AB	0	100	0

Using dataset2, the ABGD method detected a gap in the intraspecific and interspecific distances in *H1C* sequences between *M. chilensis* and *M. galloprovincialis.* The BCM and the AB methods calculated a threshold similarity value of 0.25 %, to identify barcodes as conspecific. The BCM analysis successfully assigned to the species, all the 49 individuals. However, three sequences matched with the species but with interspecific distances outside the 0.25 % threshold (MT949794, MT949825 and MT949827). Besides, the AB method was able to correctly identify the seven *M. galloprovincialis* and 39 *M. chilensis,* while three individuals were classified as “no-match”. These correspond to the same three individuals successfully identified by the BCM method outside the threshold.

### 3.3 Agreement between the HRM-PAPM method and histone H1C sequence analysis

For dataset1, the Cohen's Kappa coefficient among all SI methods ranged from zero (slight agreement) to 0.7180 (substantial agreement) (Table 4a). Moderate agreement (κ = 0.5034, p = 0.0015 and MCC = 0.5775) was obtained between HRM-PAPM and DSC methods. Substantial agreement was observed between HRM-PAPM and BCM (κ = 0.6676, p = 0.0011 and MCC = 0.6822), and between DSC and BCM methods (κ = 0.6870, p = 0.0002 and MCC = 0.7242). The AB method showed slight agreement (κ = 0) with all the other methods.

**Table 4:** Kappa concordance, confidence interval of 95%, agreement and Matthews correlation coefficient (MCC) between Species Identification methods based on the HRM-PAPM / Me15-16 and H1C barcode gene Direct Sequence Comparison (DSC), Best close Match (BCM) all barcode (AB) and Automatic Barcode Gap Discovery (ABGD) methods for **a)** dataset1(all sequences) and **b)** dataset2 (without putative hybrids).

a) dataset1				
	DSC	BCM	AB	ABGD
**HRM PAPM**	0.5034	0.6676	0	–
	(*p* = 0.00145)	(*p* = 0.00109)	(*p* = 0.5)	
	0.2516–0.7552	0.4150–0.9201	0–0	
	Moderate agreement	Substantial agreement	Slight agreement	
	MCC = 0.5775	MCC = 0.6822	MCC = 0	
**DSC**	–	0.6870	0	–
		(*p* = 0.00002)	(*p* = 0.5)	
	–	0.4848 – 0.8816	−0.1242–0.1242	
		MCC = 0.7242	MCC = 0	
	–	Substantial agreement	Slight agreement	
**BCM**	–	–	0	
	–	–	−0.0665 0.0665	–
			(*p* = 0.5)	
	–	–	Slight agreement	
			MCC = 0	
**AB**	–	–	–	–

**b) Dataset2**				

	**DSC**	**BCM**	**AB**	**ABGD**

**HRM PAPM**	1.00	1.00	0.7717	1.00
	(*p* = 0.00006)	(*p* = 0.00006)	(*p* = 0.00054)	(*p* = 0.00006)
	–	–	0.5215–1.0220	–
	Perfect agreement	Perfect agreement	Substantial agreement	Perfect agreement
	MCC = 1.00	MCC = 1.00	MCC = 0.7825	MCC = 1.00
**DSC**	–	1.00	0.7717	1.00
		(*p* = 0.00006)	(*p* = 0.00054)	(*p* = 0.00006)
	–	–	0.5215–1.0220	–
	–	Perfect agreement	Substantial agreement	Perfect agreement
		MCC = 1.00	MCC = 0.7825	MCC = 1.00
**BCM**	–	–	0.7717	1.00
			(*p* = 0.00054)	(*p* = 0.00006)
	–	–	0.5215–1.0220	–
	–	–	Substantial agreement	Perfect agreement
			MCC = 0.7825	MCC = 1.00
**AB**	–	–	–	0.7717
				(*p* = 0.00054)
	–	–	–	0.5215–1.0220
				Substantial agreement
	–	–	–	MCC = 0.7825

Excluding putative hybrids (dataset2) a higher agreement was observed, κ values ranged from 0.7717 (substantial agreement) to 1.00 (perfect agreement) (Table 4b). A perfect agreement was obtained in all comparisons among the HRM-PAPM, ABGD, DSC and BCM methods (κ = 1.00, p = 0.00006 and MCC = 1.00). The AB method showed substantial agreement (κ = 0.7717, p = 0.0005 and MCC = 0.7825) with all the other four methods.

## 4 Discussion

Molecular taxonomic identification or specimen assignment, i.e., to assign an individual to a species, is important for food safety and authenticity, wildlife forensic, conservation, property and consumers rights protection. In the food sector including, regulators, processors, retailers and consumers, there is special concern about species identification (Armani et al., 2015, Verrez-Bagnis et al., 2018). It is widely accepted the need for proper methods to support law enforcement, that could be easily implemented by private or government laboratories. However, when different analytical methods are used for the same purpose (in this case, SI) the results may not be concordant among them. Therefore, harmonization is necessary to avoid stakeholders being affected by the discrepancies among the different analytical methods (Coleman & Fontana, 2010). The harmonization among commercial methods currently used for mussel SI begins with a comparison of their performance. Discrepancies among methods could arise from the different molecular markers analyzed, but also from the sequence analysis methodology employed. In this study, we compare two mono-locus commercial methods used to identify the most traded *Mytilus* mussel species: the HRM-PAPM and the sequence analysis of the *H1C* barcode gene.

The high genetic similarity among *Mytilus* spp. has been revealed by many markers used for DNA barcoding (*COI*, *5S rDNA*, *ITS-*1, *ITS-*2 and *NTS*) (Giusti et al., 2020, Santaclara et al., 2006, Tinacci et al., 2018), which were not able to separate the different species. However, the polyphenolic adhesive protein (*PAPM*) and the histone *H1C* genes, have been shown to successfully differentiate *Mytilus* mussels (e.g., Santaclara et al., 2006, Fernández-Tajes et al., 2011, Eirín-López et al., 2002). SI results obtained by the HRM-PAPM and *H1C* gene sequence analysis showed a slight to a substantial agreement among them, using the complete dataset (dataset 1). Discrepancies between mono-locus methods are expected because they target genomic regions with different evolutionary histories (Larraín et al., 2012, Väinölä and Strelkov, 2011). The *PAPM* is a single copy gene, while the *H1* genes (including *H1C*) have evolved by gene duplication with close to one hundred copies per haploid genome in *M. galloprovincialis* (Eirín-López et al., 2002) and *M. edulis* (Drabent et al., 1999). Besides, in contrast to the highly conserved family of core histones (H2A, H2B, H3, and H4), the H1 histones are less conserved during evolution (Drabent et al., 1999).

*H1C* sequences showed very high similitude among the *Mytilus* taxa analyzed, therefore, there was no barcoding gap in the *H1C* gene to separate intra- from inter-specific sequence variation when considering all the individuals analyzed. Probably, this is the reason why FINS methodology based on the *H1C* gene was not useful for SI in smooth-shelled mussels (Fig. 1). Also, considering all the problems of tree-based identification techniques discussed by Meier et al., 2006, Collins and Cruickshank, 2013, FINS is not a recommendable approach to perform traceability in *Mytilus* species.

On the other hand, the DSC method uses a similarity criterion relying on an arbitrary threshold to consider two sequences belonging to the same species. Usually, a 1 or 2 % divergence is a reasonable rule-of-thumb in most cases, but it is prone to produce different rates of false-positive and false-negative (Collins & Cruickshank, 2013). In this study, a 1 % divergence threshold in pairwise similarities between *M. chilensis* and *M. galloprovincialis H1C* sequences, was suitable to correctly identify all samples, excluding putative hybrids (dataset2). The ABGD method was not able to identify a barcoding gap between *M. chilensis* and *M. galloprovincialis* sequences when all individuals were considered, as is in a real scenario in mussel traceability. This sequence analysis method only worked when putatively hybrids were previously excluded from the analysis. As expected, the BCM and AB methods showed a broader threshold similarity value (1.28 %) when all individuals were considered, compared with the 0.25 % threshold obtained when putatively hybrids were excluded. When these two methods were applied to identify the species in all individuals, the performance was lower. Moreover, the strictest criteria of the AB method, classified all individuals as ambiguous. It is important to consider that using the same dataset, sequence analysis methods could give different results. Therefore, the election of the sequence analysis method for SI must be considered during the standardization and validation process.

The sequences for the *H1C* gen *in Mytilus* spp. available in the GenBank, were published in two papers. Eirín-López et al. (2002) uploaded sequences from *M. edulis, M. galloprovincialis, M. trossulus, M. chilensis* and *M. californianus,* whereas Drabent et al. (1999) contributed with nine *M. edulis* sequences for this gene. Until now, the NCBI Reference Sequence Database contains no sequences for the *H1C* gen from *Mytilus* spp. The reference sequences have their provenance and validity reviewed and checked and their GenBank ID begins with NC. This quality check is extremely important to avoid taxonomic misidentifications. Reviewing the *H1C* sequences published in GenBank, we realized that the sequence AJ416422 uploaded as *M. chilensis* by Eirín-López et al. (2002) grouped with the *M. californianu*s sequence and not with *M. chilensis* (Fig. 1)*.* Besides, 27 polymorphic sites were present between our sequences of *M. chilensis* and the sequence AJ416422*,* indicating a possible error in the species assigned to the last sequence in GenBank. Mistakes uploading sequences to GenBank, especially at the species level (but not at the genus level) are common, affecting the taxonomic reliability of this database (Leray, Knowlton, Ho, Nguyen, & Machida, 2019). Both species are very different in shell morphology, *M. californianus* is a ribbed mussel, while *M. chilensis* is a smooth shelled mussel. Unfortunately, the specimen used by Erin-Lopez do not have morphological data linked to the GenBank record. The availability of reference sequences obtained from vouchered specimens in cured databases is necessary to standardize sequence-based methods as traceability tools and to avoid taxonomical uncertainties. The species from all the individuals whose *H1C* sequences were obtained in this work and uploaded to GenBank were previously checked by the 49 SNP panel (Larraín et al., 2018) and corresponded to *M. chilensis* and *M. galloprovincialis*.

Food traceability and authenticity in *Mytilus* mussels is challenging due to the natural hybridization observed in many areas where these species are cultured (Michalek et al., 2016). In the case of *M. chilensis* and *M. galloprovincialis* the presence of hybrids has been highlighted in molecular identification studies conducted on cultured specimens in Arauco and Reloncaví gulfs (Larraín et al., 2012, Larraín et al., 2019) and on exported Chilean mussel products collected from retail (Giusti et al., 2022). To overcome this challenge, new molecular techniques are necessary to separate hybrids from the parental taxa in an efficient a cost-effective way. However, according to our results, mono-locus assays are not efficient for this purpose, because they gave contradictory conclusions. Methods based on a multi-locus approach (Larraín et al., 2019) or genomic technology must be developed and validated to be used by regulatory agencies and the food industry. The higher the complexity of a method, the more time it consumes, affecting the practicability required in routine analysis. Consequently, using a small panel of informative SNPs is a good option to address the presence of hybrids (Quintrel et al., 2021). For example, all individuals used in this work were previously genotyped with a 49 SNP panel without any evidence of being hybrids. However, *H1C* sequence analysis showed strong evidence of hybridization in twelve individuals, reflecting the complexities of hybrids identification.

The SI discrepancies between these *H1C* and *PAP* genes were mainly because the putative hybrids detected by the *H1C* gene sequence analysis were not classified as such by the HRM-PAPM method, although this latter can also distinguish hybrids between these species (Jilberto et al., 2017).

The HRM-PAPM method showed total agreement with the 49 SNPs panel to identify *M. chilensis* from *M. galloprovincialis*. The *H1C* sequence analysis is helpful to differentiate between both species when the appropriate sequence analysis method is used.. Our results show that *H1C* can reveal some hybrid not detected by the HRM-PAPM, finding that is highly possible because *Mytilus* mussel hybridizes, their genome has a size of ∼ 1.6 Gb and of course we are screening only a very low fraction of it with our SI methods.

## 5 Conclusions

A high similarity of *H1C* gene within *Mytilus* spp. is evident and it hampers the differentiation between *M. edulis* and *M. galloprovincialis*. Nevertheless, the study confirms that the application of the BMC method allows both discrimination between the two species and the detection of hybrids.

Based on the *H1C* gene, the sequence analysis method affects SI outcomes. The FINS, ABGD and AB methods were not useful to identify *M*. *chilensis, M. edulis* and *M. galloprovincialis* specimens.

The different levels of agreement between the 49 SNP panel and both SI methods (HRM-PAPM and *H1C* gene sequence analysis) highlight the need for the standardization of molecular tools. The presence of hybrids is a realistic scenario in *Mytilus* mussel aquaculture that makes SI more complex. Our results indicates that analytical tools based on a multi-locus approach are needed to enface the challenge of the traceability of smooth-shelled mussels.

Each assay laboratory must decide what is the best method for mussel SI depending on the equipment available and if it can easily be combined with other methods used in the laboratory. If sanger sequencing is a routine procedure, we recommend the sequencing of the *H1C* gene followed by analysis with the BCM method. On the other hand, if the qPCR analysis is the routine procedure HRM-PAPM is the recommended method.

Our results highlight the need for standardized molecular tools to perform SI in smooth-shelled mussels, as well as the use of a multi-locus approach.

## Declaration of Competing Interest

The authors declare that they have no known competing financial interests or personal relationships that could have appeared to influence the work reported in this paper.
