# Supplementary material for: Comparison of two commercial methods for smooth-shelled mussels (Mytilus spp.) species identification
Source: Food Chem (Oxf). 2022 Jul 14;5:100121. doi: 10.1016/j.fochms.2022.100121 (PMC9294527; doi:10.1016/j.fochms.2022.100121)
Supplement: Supplementary data 1 [file mmc1.docx]

Table S1. Detailed information about mussel samples used in this study.

* Species determined with 49 SNPs panel used in Larraín *et al*. (2018).

| **Code** | **Sampling location in Chile** | **Species*** | **Lat** | **Long** | **Sampling Year** | **﻿Nº of individuals** |  |
| --- | --- | --- | --- | --- | --- | --- | --- |
|  |  |  |  |  |  |  |  |
| DICL | Dichato | *M. galloprovincialis* | -36,549 | -72,936 | 2009 | 9 |  |
|  |  |  |  |  |  |  |  |
| CNCL | Canutillar | *M. chilensis* | -41,520 | -72,338 | 2009 | 5 |  |
|  |  |  |  |  | 2013 | 5 |  |
|  |  |  |  |  |  |  |  |
| QICL | Quillaipe | *M. chilensis* | -41,549 | -72,770 | 2009 | 5 |  |
|  |  |  |  |  | 2013 | 4 |  |
|  |  |  |  |  |  |  |  |
| LACL | Caleta La Arena | *M. chilensis* | -41,683 | -72,672 | 2009 | 5 |  |
|  |  |  |  |  | 2013 | 5 |  |
|  |  |  |  |  |  |  |  |
| PICL | Pichicolo | *M. chilensis* | -42,040 | -72,591 | 2009 | 4 |  |
|  |  |  |  |  | 2013 | 5 |  |
|  |  |  |  |  |  |  |  |
| CBCL | Canal Coldita—Piedra blanca | *M. chilensis* | -43,247 | -73,695 | 2009 | 4 |  |
|  |  |  |  |  | 2013 | 3 |  |
|  |  |  |  |  |  |  |  |
| IPCL | Isla Peel | *M. chilensis* | -50,842 | -74,011 | 2009 | 3 |  |
|  |  |  |  |  | 2013 | 4 |  |
