# Supplementary material for: Comparison of two commercial methods for smooth-shelled mussels (Mytilus spp.) species identification
Source: Food Chem (Oxf). 2022 Jul 14;5:100121. doi: 10.1016/j.fochms.2022.100121 (PMC9294527; doi:10.1016/j.fochms.2022.100121)
Supplement: Supplementary data 2 [file mmc2.docx]

**Table S2**. Validation parameters of both qualitative compared methods, HRM-PAPM and Sequencing of *H1C* gene, to identify Mytilus species.

| **Parameter** | **HRM-PAPM^1^** | ***H1C* gene Sequencing^2^** |
| --- | --- | --- |
| Applicability | The method is applicable to *M. chilensis*, *M. galloprovincialis* and *M. edulis* fresh, frozen, and canned with brine, oil, hot and scallop sauce.  Interferents: vinegar (acetic acid), tomato sauce. | The method is applicable to *M. chilensis*, *M. galloprovincialis* and *M. edulis* fresh, frozen, and canned with brine, oil, hot and scallop sauce.  Interferents: vinegar (acetic acid), tomato sauce, partially in combination with analysis of other genes. |
| Practicability | Excluding the DNA extraction step, the analysis time for a minimum batch of seven samples does not exceed 6 hours, considering one analyst. Real-time PCR equipment and HRM kits are usually available in molecular analysis laboratories, and they are not dangerous for the handler. The training time of an analyst is four hours. | Excluding the DNA extraction step, the analysis time for a minimum batch of seven samples does not exceed 24 hours, considering one analyst. PCR equipment is usually available in molecular analysis laboratories and sequencing analysis can be done overnight by Sanger service laboratories. The data analysis can be done by analyst which are experienced in sequencing data analysis and sequence databases as NCBI. |
| Primer specificity | PAPM primers are specie-specific for *M. chilensis*, *M. galloprovincialis* and *M. edulis* for *in silico* and *in vitro* tests. | PCR and sequencing primers are specific for all *Mytilus species.* Discrimination of species will be done by comparison of sequence data with database entries. |
| Sensitivity (LOD) | The limit of detection (LOD) is 5 ng/μL, and the recommended DNA working concentration is 20 ng/μL DNA. | The analysis should be done with DNA extracts single mussels. Extracts from several mussels should be analyzed in parallel to exclude mixtures. |
| Robustness | The method was robust in front to slight changes in annealing temperature (58 and 60 ºC), 260/230 ratio (0.7 and 2.1), 260/280 ratio (1.7 and 2.0), annealing time (20 and 40 seconds), primer concentration (5 and 15 ng/μL), reaction volume (7 and 9 μL), and brand of HRM kit (SensiFastTM HRM Kit, Meridian Biociences® y qPCRBIO^TM^ HRM Mix, PCRBiosystems). | The analysis is performed since about 10 years in routine mussel testing and robust under controlled laboratory conditions. Limiting factor is quality and quantity of DNA, especially in canned products. |
| Sensitivity* | 0.9537^3^ | 1.0000 |
| Specificity** | 1.0000 | 1.0000 |

^1^ Data published in Quintrel *et al*. (2019).

^2^ Data provided by from Eurofins Genomics

^3^ Data obtained from Jilberto *et al*. (2017) over the analysis of 471 mussel samples.

* Estimated as the number of individuals that the HRM method correctly excluded from the species, divided by the total number of individuals who do not belong to that species.

** Estimated as the number of individuals in which the HRM method correctly identified the species, divided by the total number of individuals sampled from that species.
